# The patient experience with fatigue and content validity of a measure to assess fatigue severity: qualitative research in patients with ankylosing spondylitis (AS)

**DOI:** 10.1186/1477-7525-11-192

**Published:** 2013-11-11

**Authors:** April N Naegeli, Emuella Flood, Jennifer Tucker, Jennifer Devlen, Emily Edson-Heredia

**Affiliations:** 1 Eli Lilly and Co., Lilly, Corporate Center, Indianapolis, IN 46285, USA; 2 Oxford Outcomes, 7315 Wisconsin Avenue, Suite 250W, Bethesda, MD 20814, USA

**Keywords:** Ankylosing spondylitis, Concept elicitation, Content validity, Fatigue, Patient reported outcomes (PRO), Worst Fatigue Numeric Rating Scale (WF-NRS)

## Abstract

**Background:**

Ankylosing spondylitis (AS) is an autoimmune disorder characterized by inflammation of the spine and large joints. Fatigue is a common symptom that many AS patients find significantly impacts their health-related quality of life. The Worst Fatigue – Numeric Rating Scale (WF-NRS) assesses the severity of this symptom during the previous 24-hour period. The objective of this study was to perform qualitative research to support the development and content validity of the WF-NRS.

**Methods:**

Patients with AS were recruited from clinical sites in the U.S. for a qualitative study which first entailed concept elicitation interviews to gain understanding of the patients’ experience with AS and fatigue. Subsequently, cognitive debriefing interviews were undertaken to assess the understandability, clarity, and appropriateness from the patient’s perspective, of the content of a measure of fatigue severity.

**Results:**

Thirteen patients with AS participated in concept elicitation interviews and cognitive debriefing of the Brief Fatigue Inventory (BFI) fatigue severity subscale. The WF-NRS was developed from the worst fatigue item of the BFI as patients generally reported it to be understandable and covered an important concept, the completion instructions were modified, but the response scale remained as it was familiar and readily completed, and the recall period was appropriate.

**Conclusions:**

Patient responses resulted in the development of and supported the content validity of the WF-NRS. Further quantitative evaluation of the WF-NRS is warranted in order to assess its psychometric properties and confirm its usefulness as a clinical trial tool.

## Background

Ankylosing spondylitis (AS) is an autoimmune disorder characterized by inflammation of the spine and large joints. Prevalence rates for AS have been estimated to be 0.2 – 1.2% [1], and historically males have been found to be more predominantly affected than females [2,3]. However, this historical precedence may have contributed to gender biased research and under-diagnosis in women [4]. It is a chronic condition of young adults that commonly develops in the third decade of life [1]. Common symptoms include joint pain, fatigue, low-grade fever, loss of appetite, and eye inflammation [3]. There is wide inter-individual variability in bearing the burden of AS as some patients experience minor disabilities while others may have severe deformities of the spine. Aspects such as reduction in physical function, withdrawal from activities (including employment), and impairment in quality of life, comprise a significant portion of the disease burden [5].

Although there is variability in the burden of AS, many patients experience fatigue as part of their disease, with severe fatigue reported in 50% of patients with AS [6]. Furthermore, fatigue has been found to be one of the key symptoms that can significantly impact health-related quality of life [7], and patients with AS have reported that fatigue has impacted their social life, relationships, and work [8]. For this reason, researchers have called for both a comprehensive assessment of fatigue as part of routine clinical practice in patients with AS and the development of treatment programs directed at alleviating the fatigue that often accompanies AS [8].

The primary objectives of the qualitative research presented here are to describe the patient’s experience with fatigue with respect to their AS and to evaluate the content validity of a measure of fatigue severity for these patients. Concept elicitation interviews were undertaken to gain insights into patients’ with AS perceptions of the symptoms of their condition, especially fatigue, the importance of those symptoms, and the impacts of AS on patients’ lives. Key insights gleaned from these interviews demonstrated the importance of fatigue in AS and the need for clinical research to capture symptom severity as a means of demonstrating treatment benefit. Subsequently this research led to the development of the Worst Fatigue Numeric Rating Scale (WF-NRS), a single-item patient reported outcome (PRO) measure that assesses the level of worst fatigue severity experienced by the patient during the previous 24 hours. This manuscript presents results from qualitative analyses of the interview data to support conclusions regarding the development and usefulness of the WF-NRS for the assessment of fatigue severity among patients with AS in a clinical trial setting.

## Methods

### Study design

This was a cross-sectional, qualitative study involving one-on-one, in-person interviews. Participants were identified and recruited through three clinical sites in the U.S. with the sample size determined by saturation. Specifically, information “saturation” refers to the point in the interviewing process when interviews are no longer yielding new information and the researcher can feel confident that all important concepts related to the research question have been identified [9].

### Eligibility criteria and recruitment

Ambulatory patients with AS aged eighteen years or older were eligible for this study. Original inclusion criteria required an established AS diagnosis (modified New York Criteria) for at least twelve weeks and a Bath Ankylosing Spondylitis Disease Activity Index (BASDAI) score ≥ 4 and back pain score ≥ 4 on a 10-cm visual analogue scale (VAS). The back pain score was based upon the average of overall spinal pain and nocturnal spinal pain VAS scores for the past week. The inclusion criteria were modified after recruiting sites reported that some of the information necessary to determine eligibility under the original criteria was not readily available as it was not routinely collected or assessed in clinical practice. Specifically, the revised criteria no longer required a diagnosis based on the modified New York Criteria or a BASDAI score of ≥ 4; however, clinical confirmation of diagnosis via patient medical records was retained as a requirement. The revised criteria were adopted after recruitment of 3 participants (1 male) in order to enhance recruitment and pragmatically reflect clinical routines. Patients with complete ankylosis of the spine were not eligible.

Site staff reviewed medical charts and/or electronic patient databases in order to identify eligible participants and subsequently contacted these AS patients for further screening and possible enrollment. Institutional Review Board (IRB) approval was obtained from the Independent Investigational Review Board Inc. (Plantation, Florida, USA) prior to recruitment of participants.

### Study procedures

Before beginning the interview, the participant reviewed and signed an IRB approved consent form. Approximately one-hour long interviews were then conducted by a trained researcher who followed a semi-structured interview guide (Table 1). The first part of the interview was designed to elicit information from patients about the symptoms and impacts of AS. Participants were asked open-ended questions about their experiences with AS (e.g., “What is it like to have AS?”) and the interviewer followed up with probing questions as necessary. Then the Brief Fatigue Inventory (BFI) [10] was administered to patients after which cognitive debriefing interviews were conducted to assess the clarity, relevance, and comprehensiveness of that instrument, specifically the fatigue severity subscale. The BFI is a 9-item measure assessing fatigue severity and interference in daily life . One item from the severity subscale asks the respondent to “Please rate your fatigue (weariness, tiredness) by circling the one number that best describes your WORST level of fatigue during the past 24 hours”. Responses are on an 11-point numeric rating scale with anchors at 0 (No fatigue) and 10 (As bad as you can imagine).

**Table 1:** Example of questions in the semi-structured discussion guide

**Topic**	**Example questions for each symptom mentioned**
Description	What is it^*a*^ like? Can you describe that for me?
Frequency	How often does it happen? Does it vary? How long does it last? Does that change from day-to-day or throughout the day?
Severity	How severe is it? Does the severity change from day to day or throughout the day? What is it like when it’s at its worst? What is it like when it’s at its mildest? Does anything make it better? Does anything make it worse?
Emotional Impact	Does ankylosing spondylitis affect you emotionally? If so, how?
Impact on activities of daily living	Does ankylosing spondylitis affect your everyday activities? If so, how? Does ankylosing spondylitis affect what you do? Do you ever avoid activities because of ankylosing spondylitis?
Impact on leisure/social/family activities	Does ankylosing spondylitis affect your social activities? Does ankylosing spondylitis affect your relationships?

^*a*^*Interviewers used the term for each symptom spontaneously reported by participant.*

All interview sessions were digitally audio-recorded with the participant’s permission and subsequently transcribed for analysis purposes.

### Analyses

A thematic analytic approach was used to summarize and evaluate the data from the interviews [11]. Coding was performed using MaxQDA 10 [12], a text analysis software program designed to help organize qualitative data and allow for a thorough exploration of themes and concepts emerging from the data. After a process of independent coding of two transcripts by two team members, the team discussed and reached consensus on a codebook, which was then used by one researcher to code all transcripts.

Analysis of the cognitive debriefing portion of the interviews was on a question-by-question basis. The analysis focused on identifying any issues with the instrument with respect to clarity, interpretation, relevance, and comprehensiveness of the items, response options, instructions, and recall period. Descriptive statistics (e.g., mean, frequency) were used to characterize the demographics of the sample of participants.

## Results

A total of thirteen participants completed interviews. After the first three interviews, revisions to the inclusion and exclusion criteria (noted above) were made to enhance recruitment and the next ten participants were recruited under the revised criteria. Table 2 shows the sociodemographic characteristics of the sample. There were eight females and five males, and the mean (SD) age was 47 (13.4) years. The majority (N = 10) of participants rated their current level of AS as “moderate” and the remaining 3 participants rated their current AS level as “severe”. For the last ten participants (i.e., those recruited after the change in eligibility criteria and for whom data are available), the mean spinal pain NRS scores were: 6.0 (SD 1.2) for overall pain and 6.2 (SD 1.8) for nocturnal pain. For the entire sample, the average time since AS diagnosis was 13 years, with a minimum of 1 year and a maximum of 35 years. There were no new concepts reported or codes applied after the 7^th^ interview transcript, which suggests that saturation was attained by the eighth interview. As a means to gather a more in-depth understanding of the concept(s) being studied, we continued to interview 5 participants beyond the saturation point.

**Table 2:** Sociodemographic and Clinical Characteristics

**Characteristic**	**AS patients**	
	**(N = 13)**	
	**N or Mean**	**% or SD**
Age – years (mean; SD)	47	13.4
Sex		
Males	5	38%
Female	8	62%
Highest Level of Education Completed		
High School/GED	3	23%
Some College/Associates Degree	7	54%
College Degree	1	8%
Graduate Degree	2	15%
Current Employment Status		
Employed Full-Time	4	31%
Employed Part-Time	1	8%
Unemployed, Seeking Work	2	15%
Homemaker	2	15%
Disabled	1	8%
Retired	2	15%
Self-employed	1	8%
Racial		
White/Caucasian	10	77%
Native Hawaiian/Pacific Islander	1	8%
Other	2	15%
Ethnicity		
Hispanic/Latino	2	15%
Years Since Initial Diagnosis of AS (mean; SD)	13.0	12.3
Current Level of AS		
Moderate	10	77%
Severe	3	23%
NRS Spinal Pain Scores^a^		
Total pain	6.0	1.2
Nocturnal pain	6.2	1.8

^a^Based on final 10 participants for whom data is available.

### Concept elicitation

During concept elicitation interviews, participants reported being significantly affected by their AS with symptoms such as pain, stiffness, and fatigue/tiredness/less energy. Common impacts reported by participants associated with their condition included sleep difficulties, physical deformity, decreased mobility and activities, change in personality, frustration, realization of mortality, and uncertainty about the future. Exemplary quotes from the concept elicitation interviews are presented in Table 3.

**Table 3:** Descriptions of fatigue

**Exemplary participant quotes from concept elicitation interviews**	
**Topic**	**Quote**
*Description of fatigue*	“I do things at the same pace, but I run out of energy more quickly, which means I do a lot less things. Whereas in the beginning of the day I might be able to grab some laundry on the way and drop something off in this room and then, you know, stop and do this and stop and do that. By the end of the day it’s – the tasks are chosen carefully and they’re completed with no zest, no, it’s just done. …there’s a pretty moderate level of fatigue that I deal with. I used to have energy to go from morning till night and get everything done. Now by 5 or 6 pm I’m – at 5 or 6pm I’m basically useless…”
	“… feel tired all the time.”
	“… I feel like my life is in slow motion because I have to do everything so slow and take rest times.”
	“Once Saturday gets here, I am just wiped out. … I don’t do too much of anything on Saturdays. I mean, I just, I can’t. I sleep. …”
	“I’ve been feeling run down.”
	“It’s hard for me to concentrate; it’s hard for me to focus.”
*Clarity and interpretation of concept of fatigue*	“It’s a feeling of I’m so tired that I cannot function or anything or even think and it’s hard because there are things that I need to do.”
	(Response to probe regarding 'fatigue’ and 'tired’). “I feel like the same, almost like the same.”
	“I think fatigue, sometimes – well, fatigue to me sounds like it’s when you’re really tired, really, you know, really need to sit down or whatever.”
	“Oh, I’m always exhausted.”
	“Just – you just feel like you didn’t get a good night’s sleep and that if you have to lay down and take – to take naps, you know, to see if that would help and, nine times out of ten, I wake up just the same as I went to sleep. So it’s I can’t – I don’t really relax, I don’t think, really good 'cause I’m – you have to move around to get comfortable.”
*Frequency and duration*	“… the day when I feel more rested, on Monday, because as I say I feel like I charged my battery all weekend so I'm ready to work on Monday and as the week go on, like Tuesday morning a little bit more tired than Monday and then Wednesday, Thursday, Friday I'll finally – the last day of work, you know.”
	“I’m better in the mornings as far as being fatigued. I don’t feel fatigued when I get up in the mornings. I feel fine.”
*Fatigue and pain*	“But then, like I said, I don't know the difference any more. Like, I can't remember the difference of what it's like to not be in pain, what it's like to not actually have fatigue. So, I don't know, honestly.”
	“I think it’s that the pain kind of wears you down and I think it’s a cumulative thing.”
	“Well, it's tough because barely I work my shift. I start early in the morning and when I done – like right now I just get off from work at 2.. as soon as I get off from work I get at my home and I do nothing 'cause I'm tired, I got pain all over my body. So, I don't want to do nothing more. I just – all I think is just get home and rest. That's all I want to know. I just want to rest.”
	“… the tiredness and the pain seem to go together. When I start getting tired then my body starts aching more, you know…..”
*Other factors associated with fatigue*	“Yes, that’s a major thing. I – naps are good. I take naps all the time.”
	“.. close my eyes, just sit there and let my body regenerate.”
	“… I’ve been trying to get back on the treadmill … I was able to walk two miles last night and it took me about 38 minutes, which is not – I normally can go a little faster than that. .. I was very tired, but I felt better afterwards, …”
	“… well, to me, the reason it’s not mentioned is that everybody gets tired. It’s something everybody complains about. Tired for one reason or another. And if you talk to someone else, like I talk to my friends about being tired, and then they’ll tell you how tired they are.”

#### Description of fatigue

All thirteen participants had experienced fatigue associated with AS, although one participant was less affected at the time of the interview and another reported not currently experiencing fatigue. Three of the participants spontaneously used the term 'fatigue’. Other related terms used spontaneously, or words that were supplied in initial responses to the interviewer’s use of the term fatigue, included: tired, exhausted, feeling worn out or wiped out, (lack of) energy, run down, hard to concentrate or focus, slow motion, needing to rest, and falling asleep. Some participants reported feeling that although they did things at the same pace as before the onset of AS, they had less energy and consequently ran out of energy sooner. Others described this concept in terms of doing things more slowly, taking longer than usual to do things, or of being in slow motion.

#### Clarity and interpretation of the construct of fatigue

All participants understood the term 'fatigue’. Participants reported that 'fatigue’ and 'tired’ meant the same thing to them. One participant noted that fatigue meant being 'really tired’ while others said they felt 'exhausted’. Participants interpreted fatigue in terms of a lack of energy, running out of energy or completing tasks but without 'zest’. Some participant’s comments suggested a mental component to the concept of fatigue and, in particular, noted being 'too tired to think’.

#### Frequency and duration

Participants reported that fatigue varied over the course of the day, with several individuals reporting extreme fatigue at the end of the day. Mornings were reported to be somewhat better with respect to fatigue, with this improvement sometimes attributed to having rested during the night. Some patients, however, woke up feeling tired. It might take them considerable time to 'get moving’ in the morning, but once moving, their fatigue and pain levels seemed to decrease. For some participants, fatigue severity increased over the course of the week, and they used the weekend to rest and 'charge their battery’.

#### Fatigue and pain

Pain was another key symptom of AS, and all thirteen participants reported experiencing pain. There appeared to be a complex relationship between fatigue and pain. For instance, when asked about fatigue, participants would often respond by talking about their pain. However, it was not possible to identify either a consensus on the participants’ perceived association between fatigue and pain or a causal relationship between the two. Some participants reported being 'tired from the pain’, suggesting that the experience of pain was physically and/or emotionally tiring. Conversely, others reported that when they were tired, more pain was experienced. Eight participants reported experiencing disturbed sleep because of pain during the night, and the tiredness in the morning was seen as result of this pain.

#### Other factors associated with fatigue

Some participants reported feeling depressed. The experiences of both pain and fatigue could be related to, or exacerbated by, feelings of depression. Other sleep disturbances (i.e., those not related to pain) were also reported as contributing to waking up tired. Participants reported that resting and taking naps could help to reduce fatigue.

### Cognitive debriefing

Overall, the cognitive debriefing interviews revealed that participants with AS interpreted the items of the BFI in a consistent manner and, based on the judgment of the study team, as intended. Table 4 presents exemplary quotes from the study participants during the cognitive debriefing interviews.

**Table 4:** Content Validity of the WF-NRS

**Exemplary participant quotes from cognitive debriefing interviews**	
**Topic**	**Quote**
*Understandability*	If I’d had any worse in the last 24 hours, which last night was one of those nights that wasn’t a good night and I think I was up four or five times, you know?
	“ … there’s just that little period once I’ve been home for a half hour, 45 minutes and then after I’ve been home for, you know, over an hour, it’s just – that’s when I’m the most tired and then I kind of get my second wind… So I’d say it’s just that period after work where I move up to about a six.”
	“So if you want to ask me what my worst level of fatigue is, maybe – again, I don’t – these were just all kind of – I always think if they’re trying to get a trick question out of me, so “Best describes your worst level of fatigue during the past” – I don’t know. That worst kind of throws me off by that. Because isn’t fatigue bad anyways, no matter how you look at it?
*Response scale*	“How did I decide? Wait a minute. No fatigue. Oh, I meant to do it the other way. I really meant the other way. So that really should have been at this end…Oh, ok. I’m sorry I misread that one. I would think around a 7. It’s, you know, it’s as bad as you can imagine. I would think maybe an 8. Can I change it?
*Recall period*	“… Depending on what the person is doing every 24 hours – at least for me every 24 hours is different… so I understand that you’ve got to put a time on the stuff, but the last 24 hours for me is different than the middle of the week 24 hours of the last week.”
	“Yeah, I think that’s, kind of – I probably should do one of these every day, that way I would know exactly how I feel every day, you know, because it just makes you realize just how tired you are or how it affects your daily life, you know, so, very interesting.”
	“For me, I – 24 hours, it depends on what – for me, I would look at a week, because I think during the course of a week you will see – there’s a – and I don’t know how everybody else is, but I just know during the course of a week there could be a week that’s good, there could be a week that has a day or so that’s not so good, you know, and that’s just…: I just think you’ll get a bigger picture by looking at it over a course of a week.”
*Possible mediating factors and adjustments*	“I haven’t felt like I’ve rested well.”
	“That was just I pretty much wore myself out, just of the things that I did and the fact that I think my body doesn’t work as fast, you know, there’s always the pain and the stiffness, so therefore, to me, you know, it’s – that causes the fatigue to really, you know, get worse, so…I just thought to myself that that’s just how I felt, that I just really, you know – I really don’t know how to explain it. It’s not that I just want to you know, lie down and sleep all the time. It’s just that you just feel like you never – with the stiffness and the pain it just never gives you a feeling of you’ve really had a good rest. You’ve really had a good, you know, it’s just really hard to, and that’s why I put eight because that’s what I felt, you know, I haven’t felt like I’ve rested well, you know, so, I don’t know.”
	“Now 10 is almost like crawling into bed and, you know, and listening to something and having a difficult time just filtering and, you know, and just – that’s severe. And what’s amazing is that – I don’t want you to think that there is, you know, it’s – it’s not a mental problem. It’s like you’re so fatigued that it’s just difficult to process the info, you know, I dunno.”
	“When my mother came into my room I actually snapped at her, you know? I didn't mean to and I felt really bad.”
	“… because you do kind of adjust 'cause in your mind you do think, what – and that’s why, I guess, I go back to that day that I say that was my worst day because in my mind I can kind of know what I’ve done …”

#### Understandability

In general, participants found the worst fatigue item of the BFI to be clear and easy to understand. They understood that the item was asking them to rate the severity of their fatigue at its worst level over the previous 24 hour period. However, two issues related to comprehension were reported during the interviews. First, one participant noted some confusion with the wording of the item since both 'best’ and 'worst’ appeared together in the item (i.e., 'choose the one number that best describes your WORST level of fatigue’). Second, one participant was unfamiliar with the term “weariness”, which appears in parentheses next to the term “fatigue” to further define it. Furthermore, no participant spontaneously used the term 'weariness’ or feeling 'weary’ during the concept elicitation part of the interviews. Three participants used the term only after seeing it in the BFI. The remaining participants in the sample either did not use the term at all or referred to a symptom that 'wears on you’, or 'wears me out’, or 'wears me down’.

#### Response options

The response scale used in the worst fatigue item was generally well understood. Some participants found the eleven-point numeric rating scale to be similar to other scales they had used previously. There was one participant who initially answered with a '2’, but when the interviewer asked how she had decided upon her answer, the respondent realized she had interpreted the direction of the scale incorrectly and corrected her response to an '8’. Aside from that oversight, no other difficulties with the response scale were reported, and the other participants found the scale to be clear and appropriate.

#### Recall period

The recall period of the worst fatigue item was considered appropriate, and participants appeared to have no difficulty recalling their experiences with fatigue over the past 24 hours. While most participants used the recall period accurately in answering the item, there was evidence that one patient may have used a longer recall period in responding, as when asked to define the item, the participant described fatigue experienced a few days prior. In addition, some participants suggested increasing the recall period to three days or a week because they were concerned that a 24 hour recall period might not provide an accurate picture of their fatigue, especially given the variability of this symptom.

#### Possible mediating factors

During the cognitive debriefing interviews, the replies of some of the participants indicated that they had taken other factors into account when selecting the response that reflected their worst fatigue severity. Some participants made an initial rating, as described above, and then adjusted the reported score if they could identify a specific reason for fatigue severity. For instance, participants reported adjusting a rating downward if there were circumstances that might explain their fatigue, such as if they had been particularly active. Other reasons included the effort required to do tasks, rest and/or sleep, lack of energy, irritability, cognitive impairment, and pain.

### Recommended changes

Based on the participants’ comments during the interviews, several recommendations for modification were proposed for clarifying the verbiage of the worst fatigue item of the BFI and, also, for its administration in clinical trials, which led to the development of the WF- NRS “Please rate your fatigue (feeling tired or worn out) by circling the one number that best describes your WORST level of fatigue during the past 24 hours”. Responses are on an 11-point numeric rating scale with anchors at 0 (No fatigue) and 10 (As bad as you can imagine).

First, in order to enhance understandability, the word 'best’ was removed from the instruction component of the item. Second, the term 'weariness’, which was used to elaborate on the term 'fatigue’, was replaced by the term 'worn out’ which seemed to resonate with a larger number of participants.

Third, in order to better reflect the variability of fatigue severity found in the AS population, it was recommended that the fatigue severity measure be administered as a daily diary in the clinical trial setting. Finally, a last recommendation was to include in the AS clinical trials items or PRO tools that would capture potential covariates of fatigue, such as sleep disturbance, physical activity level, and pain.

## Discussion

In this study, we intended to capture participant’s experience with having AS. Many were young adults (the youngest was 26 years old) or had lived very active lives (the oldest was a 76-year old competitive triathlete) prior to disease onset. Fatigue and pain were often experienced at extreme levels, and the symptoms affected all areas of participants’ lives: mobility, work, career aspirations, social activities, relationships, and emotional well-being. Although participants typically mentioned chronic, and sometimes excruciating, pain when first asked about their experience of AS, when probed about fatigue, they responded with 'Oh yes, very much so’ and 'Oh, I’m always exhausted’. One participant suggested that one possible explanation for not reporting fatigue spontaneously could be the 'normalization’ of the phenomenon by themselves (i.e., feeling fatigued becomes customary) or others (i.e., 'everybody gets tired’), and hence participants’ omission to report this. Fatigue severity was reported to be highly variable from day-to-day as well as throughout the day, with the evening often cited as the time of worst fatigue severity. Participants mentioned a number of factors influencing fatigue severity, including pain, sleep disturbances (that may be related to pain), exercise or activity, and feeling depressed.

Results from the concept elicitation interviews also indicated that all participants clearly understood the term 'fatigue’ and some of them used it spontaneously when describing their condition. It is possible that individuals with AS quickly learn the term because it is used by doctors and other patients when discussing the condition. While participants also used other terms and phrases to describe fatigue, such as tiredness, feeling worn out, exhausted, needing to rest, slowed down, etc., the term 'fatigue’ resonated with participants and was considered clear and appropriate for describing how AS makes them feel.

In summary, the concept elicitation interviews confirmed that fatigue was a key symptom of AS. All participants reported experiencing fatigue at some point during their illness, with all but one participant currently experiencing fatigue. Along with pain, fatigue was considered a bothersome symptom of AS, with one participant reporting that fatigue was the most bothersome symptom.

Although there have been a large number of scales developed with the intent of measuring fatigue [13], the WF-NRS has a number of characteristics that distinguish it from the alternatives. First, the WF-NRS was developed based on qualitative input (concept elicitation interviews and cognitive debriefing of the BFI) from an AS population ensuring the assessment of an important and relevant aspect of fatigue severity. This type of patient input is a key aspect for establishing content validity as the fatigue experience may differ across patient populations and scales developed for one condition may not be appropriate for use in another. Many of the existing fatigue scales were developed in patient populations other than AS, such as those with cancer [10,14,15], chronic fatigue syndrome[16-18], multiple sclerosis [19-21], rheumatoid arthritis [22], or general medical patients [23,24]. To the best of our knowledge, content validity in AS has not been established for most of these instruments. Second, the WF-NRS is a single-item, unidimensional instrument inquiring about the worst level of fatigue in a 24 hour period as a measure of fatigue severity, which the interviews revealed to be relevant and understandable in patients with AS. Having respondents answer queries based on their worst experience is consistent with recommendations from PRO development guidelines for using appropriate methods and techniques to enhance the validity and reliability of self-reported data [25]. In contrast, the other measures listed above are all multiple-item tools that assess fatigue as either a unidimensional [10,14,16,19] or a multidimensional [15,17,18,20-24] construct. The brevity of the WF-NRS may make it particularly suitable for use in clinical trials with a patient population that suffers from fatigue, especially if the trial subjects will be expected to complete a battery of other assessment instruments. Finally, the 24-hour recall period appears to be short enough that patients will not have difficulties in accurately completing the instrument. The study participants suggested that fatigue severity was periodic and therefore other recall periods, such as “at the present time”, may not capture clinical peaks. Additionally, capturing the worst level of fatigue in a short timeframe does not require the patient to average his or her symptom severity over time. Patient responses that rely on memory based on long periods of time, or require the responder to average their response, may introduce recall bias [25]. Moreover, incorporating the WF-NRS in a daily diary may help ensure that day-to-day variability in worst fatigue could be readily captured and addresses participants’ concerns that just one assessment may not accurately characterize their fatigue severity. The addition of a clinical outcome assessment tool to capture cognitive impairment should also be considered as participant comments suggest a mental component of fatigue presenting as a consequence of AS.

A limitation of the study may be that the small sample size allowed participant’s input to be obtained from only three clinical sites, although they were located in separate regions of the country. However, the intention of sampling for qualitative research for the development of a patient-reported outcome measure is not to obtain a representative sample of the epidemiologic profile of the patient population but rather to ensure an enriched diversity in patient and disease characteristics, and the distribution of variables indicated a diverse sample with respect to sociodemographic characteristics was obtained. Also, the recruited participants did not include AS patients with a mild level of severity. While the results of this initial qualitative study of fatigue in AS patients were promising, further quantitative research is indicated. In particular, additional research with the WF-NRS is required in order to assess its test-retest reliability; to examine its concurrent, discriminative, and construct validity; and to determine its sensitivity to change. Additionally, the translatability of the WF-NRS into other languages and cultures would also need to be examined before using the instrument in global clinical trials. Nonetheless, the interviews completed with patients with AS in the present study led to the development of the WF-NRS and ensured that the content was relevant to the AS population and the clarity, interpretation (as intended per judgement of the study team), response scale, and recall period were all appropriate.

## Conclusion

The reported prevalence and bothersomeness of fatigue supports the importance of assessing the concept of worst fatigue severity in patients with AS to capture treatment benefit(s) in a trial setting. Results of concept elicitation interviews and cognitive debriefing of the BFI fatigue severity subscale support the development and content validity of the Worst Fatigue – Numeric Rating Scale to assess that aspect of fatigue. Additional research is warranted to further evaluate the psychometric measurement properties of the instrument, including construct validity, reliability, and sensitivity to change, and, thereby, support its use in clinical trials.

## Abbreviations

AS: Ankylosing spondylitis; BASDAI: Bath ankylosing spondylitis disease activity index; BFI: Brief fatigue inventory; NRS: Numeric rating scale; PRO: Patient reported outcomes; VAS: Visual analogue scale; WF-NRS: Worst fatigue - numeric rating scale.

## Competing interests

ANN and EEH are fulltime employees and hold stock shares and/or stock options with Eli Lilly and Company. Eli Lilly and Company is investigating new pharmaceutical therapies for the treatment of ankylosing spondylitis.

## Authors’ contributions

All authors were responsible for study design, data analysis, data interpretation, and manuscript writing. All authors read and approved the final manuscript.
